# Characteristics and motivations of volunteers providing one-to-one support for people with mental illness: a survey in Austria

**DOI:** 10.1007/s00127-018-1514-1

**Published:** 2018-04-12

**Authors:** Gϋnter Klug, Sarah Toner, Karin Fabisch, Stefan Priebe

**Affiliations:** 1 Psychosocial Services, Society for Mental Health Promotion, Graz, Austria; 2 Unit for Social and Community Psychiatry (WHO Collaborating Centre for Mental Health Service Development), Newham Centre for Mental Health, Queen Mary University of London, London, UK; 3 University Hospital for Psychiatry and Psychotherapeutic Medicine, Medical University Graz, Graz, Styria Austria

**Keywords:** Volunteer, Befriend, Motivations, Mental illness

## Abstract

**Purpose:**

Large numbers of volunteers provide one-to-one support for people with mental illness, sometimes referred to as befriending. However, there has been very little research on their characteristics and motivations. This study aimed to assess the personal characteristics and motivations of such volunteers across different regions in Austria.

**Methods:**

Questionnaires assessing characteristics and motivations were distributed to 663 volunteers providing befriending for people with mental illness within volunteering programmes organised in four Austrian regions.

**Results:**

Questionnaires were completed and returned by 360 out of 663 approached volunteers (response rate 54%). Whilst most socio-demographic characteristics were widely distributed, 78% were female; 42% reported to have a family member and 56% a friend with a mental illness. Most volunteers cited motivations to do something both for others (e.g. “feel a responsibility to help others”) and for themselves (e.g. “enhance my awareness of mental health issues”). When the total group was divided into four subgroups in a cluster analysis based on their socio-demographic characteristics, a subgroup of female, single and younger volunteers in full-time employment expressed motivations to achieve something for themselves significantly more often than other subgroups.

**Conclusions:**

The study provides the largest sample of volunteers in befriending programmes for people with mental illness in the research literature to date. The findings suggest that people with different characteristics can be recruited to volunteer for befriending programmes. Recruitment strategies and supervision arrangements should consider motivations both to help others and to achieve something for themselves, and may be varied for specific volunteer subgroups.

## Background

People with severe mental illness have smaller social networks and report substantially higher levels of social isolation and loneliness than the general population [1–3]. As isolation and lack of social support are associated with poorer outcomes [4], attempts should be made to provide more social contacts and supportive relationships for people with mental illness.

One option to help people with mental illness to overcome isolation and link them with the local community is through volunteers, who spend time with people with mental illness and engage in a range of activities. Volunteers are unpaid and invest their free time to offer support. The willingness to help and spend time with people with mental illness is inconsistent with the reported tendency to distance oneself from individuals with mental illness, as found in a systematic review of public attitudes toward mental illness [5]. The input of volunteers may help people with mental illness, provide positive experiences for the volunteers and be a means to reduce marginalisation and increase social cohesion.

One form of volunteering is the provision of regular one-to-one contacts, which is often referred to as befriending. The term befriending has been criticised as potentially misleading, but is widely used in the literature [6]. Although the evidence on the effectiveness of befriending on patient outcomes remains limited and inconsistent, reviews suggest that it can reduce depressive symptoms [7], social anxieties and isolation [8, 9], and be helpful for patients with a range of mental and physical conditions [10]. It has been implemented in health and social care settings across Europe, North America and Australia [11–16]. Usually, the aim of befriending programmes is not to replace professional treatment and care, but complement what professionals can do and use a more personal, informal and social approach to support patients. Yet, although not replacing professional input, it is possible that effective befriending may relieve pressure on mental health services [9, 17].

Despite the wide use of befriending programmes and their great potential for strengthening the social integration of people with mental illness, little is known about those who volunteer as befrienders. Very limited research has been conducted to assess who these volunteers are and why they spend their free time to help people with mental illness. A systematic review on volunteers in mental health care found data on some characteristics of a total of 540 volunteers across 14 publications [18]. Of these, only 69 volunteers participated in befriending as part of their volunteering role, and data on these 69 volunteers originated from five different publications. Thus, the existing literature provides little data and is based on small studies.

More detailed information on the socio-demographic characteristics and motivation of volunteers appears essential for tailored recruitment initiatives and for improving the programmes. The motivation of volunteers drives the initial decision to engage in a programme [19], and also influences the longevity of the commitment [20]. Moreover, a review of practices in organisations running general volunteering programmes, i.e. beyond mental health care [21], and models on volunteering motivations suggest that the approach, behaviour, experiences and satisfaction of volunteers vary depending on their motivation. Thus, organisations in charge of volunteering programmes and mental health services should understand the motivational patterns of volunteers to maximise the potential of befriending as a resource.

Against this background, we conducted a survey among volunteers providing some type of befriending to people with mental illness in Austria. We addressed three research questions: (1) what are the socio-demographic characteristics of the volunteers? (2) What are their motivations? and (3) are there different subgroups of volunteers according to their socio-demographic characteristics and, if so, do their motivations for volunteering vary?

## Method

This was a cross-sectional survey in five volunteering organisations (‘gemeinnützige Vereine’) in Austria that run volunteering programmes with befriending. We collected self-reported data from volunteers about their socio-demographic characteristics and motivations, using a specifically designed questionnaire. Ethical approval was granted by the Medical University of Graz (ref: 26–319 ex 13/14).

### Eligibility criteria

We included all current volunteers of the five participating organisations providing some type of befriending to people with mental illness.

### Recruitment

Five major volunteering organisations in Austria were approached to participate in the survey. They were the “Gesellschaft zur Förderung seelischer Gesundheit” (society for mental health promotion), the “Verein pro humanis” (both covering the region of Styria), “pro mente Kärnten (covering Carinthia) “pro mente Ober-Ӧsterreich” (covering Upper-Austria) and “pro mente Wien” (covering Vienna), thus organisations covering four of the eight Austrian regions and the capital Vienna. The organisations, each with up to 1500 volunteers, are united in the umbrella organisation “pro mente Austria”, which is responsible for most of the Austrian non-profit (‘gemeinnützige’) organisations in mental health care and psycho-social services, and therefore, share similar procedures despite practicing across differing regions. These include training volunteers, matching each volunteer with a client (an individual with mental illness) to meet for 1–2 h weekly and regular supervision of volunteers to offer support, if needed. Each organisation was contacted by the first author who explained the aims of the project and obtained consent and practical support. The participating organisations then distributed the questionnaires, information sheets and consent form to all 663 current volunteers providing befriending to people with mental illness.

### Materials

The questionnaire was developed through a collaborative and iterative process between the research teams in Austria and London. The question and answer categories were based on a wider research programme on befriending for patients with mental illness conducted at the Unit for Social and Community Psychiatry at Queen Mary University of London [6, 18, 22]. They reflected themes identified in a systematic review of volunteering in mental health settings [18] and were further refined in a consultative process involving experts working in volunteering organisations in Austria. The English items were translated into German by bi-lingual researchers. The final questionnaire comprised simple and multiple-choice questions on socio-demographic characteristics, experiences of mental health treatment, and motivations to volunteers. The questions on motivations required yes/no answers to predefined categories.

### Procedure

Distribution of the information sheet, questionnaire, consent form, and return envelopes was conducted by the five volunteering organisations. All questionnaires were marked with an identification code matched to the volunteer by their organisation, the list of matched volunteer names and codes was held by the relevant volunteering organisation. Coded envelopes and questionnaires were sent by participating volunteers directly to the study director. Therefore, the volunteering organisation had no access to completed questionnaires and the study director was unable to determine the identity of respondents. Further, if the study coordinator had required details of a participant’s identity, the volunteering organisation was required to gain written consent from the relevant volunteer before it is released. At no point during the investigation did the study investigator require participants’ information, therefore, all participants remained anonymous. Data were collected over a 12 months period, and subsequently entered into SPSS (version 24) for analysis.

### Analysis

Demographic characteristics and motivations were assessed using descriptive statistics. To form subgroups of volunteers, the socio-demographic characteristics were used in a two-step cluster analysis approach, considering the sample size and the combination of continuous and categorical demographic variables measured. K-means clustering was used to determine the appropriate number of clusters, followed by a hierarchical agglomerative procedure in the second step [23]. The final clusters were determined by the gender, employment status, marital status and age of volunteers, in order of relative contribution to the cluster formation.

Clusters were then used as an independent variable in a Chi-square analysis of the “yes”/“no” responses to the motivational questions to explore whether motivations varied significantly across subgroups. Due to the explorative nature of the analysis, no adjustment was made for multiple testing. In the case of significant differences across groups, residuals were examined to identify specific subgroups accountable for group-level differences. Standardised residual values greater than 1.96 and 2.56 were considered to indicate significant and highly significant relationships, respectively.

## Results

Completed questionnaires were returned by 360 volunteers, which is a response rate of 54%. Given the population size, the percentages identified in this survey can be regarded as accurate with a 3.5% margin of error at the 95% confidence level.

### Characteristics

The socio-demographic characteristics of the sample are shown in Table 1. The sample was predominantly female (78.8%), with a mean age of 54.5 years (SD = 13.0). Most of the volunteers reported experiences with people with mental illness, either as family members (42.0%) or friends (56.0%). Whilst 34.9% had been in some form of mental health treatment in the past, this applied to only 7.6% at the time of the survey.

**Table 1:** Socio-demographic characteristics of sample

Characteristics	Frequency (*n* = 360)
Median age (mean, SD)	56.0 (54.5, 13.0)
Marital status (%)	
Single	16.6
Married	48.6
Divorced	14.4
Widowed	6.5
Civil partnership	9.3
Other	3.7
Living situation (%)	
Alone	31.0
With partner	42.7
With family	20.9
Group housing	3.4
Other	2.0
Income (%)	
Employed	44.7
Unemployment benefits	11.5
Regular pension	36.2
Disability pension	3.7
Other	4.0
Close friend with mental illness (% yes)	56.0
Family member with mental illness (% yes)	42.0
Past mental health treatment (% yes)	34.9
Current mental health treatment (% yes)	7.6

### Motivations

The first column in Table 2 shows the motivations as rated by the total sample of the volunteers. Five motivations were rated by more than half of the sample. They include three motivations that focus on what the volunteer intends to do for others (“give something back”, “feel a responsibility to help others”, “helping others is part of my philosophy of life”) and on what the volunteer may achieve for him/herself (“enhance my awareness of mental health issues”, “acquire new skills”).

**Table 2:** Percentage of the total sample and each of four subgroups stating the motivations for volunteering

Motivation	Total	Subgroup			
		1 *N* = 95	2 *N* = 75	3 *N* = 88	4 *N* = 77
Feel responsibility to help others	72.5	78.9	69.3	73.9	66.2
Helping others is part of my philosophy of life	65.1	70.5	62.7	69.3	55.8
Enhance my awareness of mental health issues^1^	61.2	60.0	50.7	59.1	75.3*
Acquire new skills	56.1	56.8	56.0	52.3	59.7
Give something back	50.4	47.4	64.0	43.2	49.4
I wanted to do something useful with my spare time^2^	33.7	45.3	38.7	21.6*	28.6
Meet new people	22.7	28.4	22.7	14.8	24.7
Curious to find out if I am suitable for the role	20.6	24.2	13.3	19.3	24.7
To gain psychologically relevant experience (e.g. for my career)^3^	20.3	9.5*	10.7	22.7	40.3**
To feel needed and acknowledged^4^	19.4	26.3	22.7	18.2	9.1*
Helping others is part of my religious belief^5^	16.4	21.1	16.0	20.5	6.5*
Test out career aspirations^6^	12.5	4.2*	8.0	13.6	26.0*
Find explanations for my own behaviour	9.9	13.7	8.0	6.8	10.4
Because of a recommendation of somebody in the mental health/social work field	6.6	7.4	8.0	4.5	6.5
Have close contact with others	6.3	8.4	6.7	2.3	7.8
“Befriending” looks good on my CV^7^	4.5	2.1	0.0	4.5	11.7**
To feel like a better person	3.9	5.3	6.7	1.1	2.6
To be accepted and liked	3.0	6.3	2.7	1.1	1.3
I have received voluntary help in the past, and wanted to give something back	2.1	2.1	0.0	1.1	5.1

^1–7^Items that significantly related to subgroup membership: ^1^*X*^2^ (3) = 10.20* p* = .017, ^2^*X*^2^ (3) = 12.88,* p* = .004, ^3^*X*^2^ (3) = 30.47,* p* < .001, ^4^*X*^2^ (3) = 8.74,* p* = .033, ^5^*X*^2^ (3) = 8.07,* p* = .045, ^6^*X*^2^ (3) = 20.19,* p* < .001, ^7^*X*^2^ (3) = 15.01,* p* = .002

*Significantly different from expected value at 5% level

**Significantly different from expected value at 1% level

### Subgroups

The cluster analysis based on socio-demographic patterns yielded four subgroups with an acceptable level of cohesion and separation. The clusters—defined by their gender, income, marital status, and age—are summarised in Table 3. The motivations for each of the four subgroups are also shown in Table 2.

**Table 3:** Characteristics of four volunteer subgroups

Volunteer subgroup	Gender	Employment status	Marital status	Median age (mean, SD)	Proportion of sample, *N* (%)
1	Female	Retired	Married	64.0 (64.5, 6.0)	95 (28.4)
2	Male	Retired	Married	58.0 (58.1, 10.6)	75 (22.4)
3	Female	Full-time	Married	51.0 (51.9, 9.7)	88 (26.3)
4	Female	Full-time	Single	44.0 (41.8, 13.1)	77 (23.0)

Most of the 19 pre-defined motivations did not significantly differ across the subgroups. Yet, seven motivations did show significant variations, and in five cases the significant difference was between the fourth subgroup and the others. As compared to the total sample, that subgroup of younger women in full-time employment wanted more often to enhance their awareness of mental health issues, gain psychologically relevant experience (e.g. for their career), and test out career aspirations. They also rated more frequently that befriending would look good on their CV. At the same time, the feeling to be needed and acknowledged and a drive to help others as part of a religious belief were less important to them relative to other subgroups.

## Discussion

### Main findings

This large survey of volunteers who befriend patient with mental illness shows that volunteers may have very different socio-demographic characteristics, but tend to have had contact with people with mental illness before, either in their families or as friends. A substantial proportion, although a minority, also had some mental health treatment themselves, but are not currently in treatment. The majority expressed motivations to do something both for others and for themselves. There are subgroups of volunteers who differ in their socio-demographic characteristics. Motivations to volunteer are largely similar across the four subgroups, but for one subgroup of younger and female professionals, motivations to achieve something for themselves appear relatively more important than for other subgroups.

### Strengths and limitations

The study is—to our knowledge—by far the largest survey of volunteering befrienders for people with mental illness that has hitherto been available in the scientific literature, and the response rate of 54% may be seen as relatively good for surveys with a postal return of questionnaires [24]. The sample size was large enough for an exploration of subgroups through a cluster analysis, an approach that had not been possible with previously available data. Another strength is that the survey was conducted in different regions of Austria, including the capital Vienna and rural areas. Thus, the results do not depend on the specific context of one region or on the potentially distinct conditions across urban and rural areas.

The main limitation of the study is that the response rate of 54%—although good for this type of surveys—still means that 46% of volunteers did not respond. Procedural approaches ensured that volunteers remained anonymous to the study investigators, thus rendering it unlikely that volunteers would avoid responding truthfully, or at all. Despite this, it is impossible to assess whether the response rate has impacted on the findings and, if so, how.

In the interpretation of the findings, any generalisation to the situation in other countries should be done with caution. Before similar studies have been conducted in other countries, it is difficult to say to what extent the characteristics and motivations of volunteers may vary across different national contexts. One should also consider that the German term for volunteer (‘ehrenamtlich’) has a slightly different connotation than the English “volunteer”, corresponding more closely to “honorary officer” status. Again, in the absence of further data, it may be impossible to assess whether such terminological aspects influence who volunteers for befriending programmes and why.

### Comparison with existing literature

#### Characteristics

The breadth of volunteer characteristics such as age, employment status, and marital status corresponds with the range of characteristics found by Hallett and colleagues [18] in their systematic review. This consistency may indicate some generalisability of the present findings, given that the data collected previously comprised British, German, American and Swedish samples [18].

#### Motivation

Substantial research has been conducted on the motivations underpinning volunteering behaviours in general. For instance, the Volunteer Process Model (VPM) by Omoto and Snyder [25] proposes key domains of motivations, including values of altruism and humanitarianism, career prospects, development of social ties, and gaining knowledge and skills. While the motivations rated in the current study do not mirror the model directly, most of the motivations expressed in this survey match on to the domains of the VPM.

The findings of this survey are also largely consistent with data from previous investigations of motivations of volunteers, not specifically befrienders. Anderson and Moore [26] reported that the majority of volunteers did so to help others (75.1%) or to feel useful and needed (50.6%). The former closely reflects the 72.5% response rate to “I feel a responsibility to help others” in the present findings. Whilst only 19.4% in this survey stated that they were motivated by a desire “to feel needed and acknowledged”, other similarly “altruistic” motivations were rated more frequently. As the analysis of subgroups in this study has shown, motivations can vary across groups, and the overall percentage of volunteers stating a specific motivation may well be influenced by which type of volunteers were interviewed.

Previous reports have emphasized the importance of religious beliefs and values in determining altruistic or voluntary behaviour [27]. Snyder and DeBono have termed this the “value-expressive function”, and it has been postulated that voluntary activity enables the individual to act on their underlying values, and therefore, demonstrate a key element of their identity [28]. This concept has also been extended to suggest that volunteering may represent a means by which people may pass their religious beliefs or personal values on to others. While only 16.4% of the total sample selected “Helping others is part of my religious belief”, the response was found to vary significantly depending on subgroup membership. At the same time, the related motivation “Helping others is part of my philosophy of life” was one of the most frequently selected items (65.1%), with no statistical variation across clusters. The current findings, therefore, lend a degree of support to assertions of the “value-expressive function”, emphasising more general values rather than specific religious beliefs.

Other motivation groups focus on the needs of the volunteering individual rather than for the benefit of others. Motivations such as ““Befriending” looks good on my CV”, “To gain psychologically relevant experience (e.g. for my career)” and “Test out career aspirations” may be considered under the “career prospects” values within the value-expressive function. While these “egoistic” motivations have been linked with less sustainable volunteering activity than altruistic goals [29], it is worth noting that the subgroup linked with such motivations also had a higher rate of endorsement for motivations such as “I have received voluntary help in the past, and wanted to give something back”. The findings, therefore, suggest a greater complexity in motivations to volunteer beyond that of altruism and what may be seen as egoism; personal history and experience may warrant further attention in future consideration of motivations. Further, while such career-centred motivations may indicate shorter term voluntary roles, they are also indicative of an intention to engage in similar roles as part of a future career. As such, services may benefit in the long-term by encouraging and catering to the motivations of these volunteers.

### Implications

The findings may have implications for the practice of volunteering in mental health care and for future research.

The data on socio-demographic characteristics suggest that volunteers for befriending programmes can come from different population groups and that a narrowly defined typical volunteer for such programmes does not exist. Thus, recruitment initiatives may approach wide parts of the population, probably with the expectation to recruit more women than men.

Whilst motivations also vary, most volunteers express motivations to do something for others as well as motivations to achieve for themselves. Both motivations are obviously legitimate and need to be considered when recruiting and supporting volunteers. As reported by Clary and Snyder [30], advertised volunteering roles were considered more persuasive when the information conveyed was congruent with the motivations deemed “most important” by targeted groups. Whilst recruitment in general can appeal to a range of motivations, more targeted strategies may be used when addressing a subgroup of younger professional women who are interested in using the experience of befriending for their progress and career. For example, specific advice can be provided for how to use the experience as a befriender to shape and support career aspirations. And the programmes themselves, including the supervision and support arrangements, can be designed to maximise the benefit for the volunteers in line with their motivation, be it to satisfy their intention to help others or to meet their wish to further their career. This motivation-oriented approach may have implications particularly for those with “egoistic” motivations. Such motivations have been linked with shorter periods of commitment to voluntary roles relative to altruistic motives [29].

For research, this survey can only be a further step towards more systematic and detailed studies about the characteristics, motivations, experiences and outcomes of volunteers in befriending schemes. Similar surveys like the presented one should be conducted elsewhere to explore the extent to which different contexts impact on the approach, viability and effects on befriending programmes. One might even consider establishing routine documentation systems to collect some of these data, although such data collection can be difficult in volunteering organisations. Future research may complement cross-sectional surveys by in-depth qualitative explorations, longitudinal studies and preferably randomised controlled trials to assess the benefits of befriending programmes both for people with mental illness and the volunteers, and potentially also for the communities in which they live.

## Conclusion

Many volunteers across the world invest their free time, energy, and good will to help people with mental illness. In some cases, volunteers regularly meet with the person with mental illness through befriending programmes, offering one-to-one support through talking or initiation of joint social activities. They provide a type of input and help that professional services would and could not deliver. As such, these volunteers deserve for any potential benefits of volunteering to be maximised. This may be facilitated using data collated by the research community. Yet, the interest in further research goes beyond this, as volunteering programmes—such as befriending or in other forms—have great potential to link people with mental illness with other parts of their communities. This survey has shown, that—for utilising that potential—wide parts of the population may be approached to engage in such programmes and that, for most volunteers, motivations both to help others and to achieve something for themselves need to be considered.
